# Tiling Histone H3 Lysine 4 and 27 Methylation in Zebrafish Using High-Density Microarrays

**DOI:** 10.1371/journal.pone.0015651

**Published:** 2010-12-20

**Authors:** Leif C. Lindeman, Andrew H. Reiner, Sinnakaruppan Mathavan, Peter Aleström, Philippe Collas

**Affiliations:** 1 Faculty of Medicine, Institute of Basic Medical Sciences, University of Oslo, and Norwegian Center for Stem Cell Research, Oslo, Norway; 2 Stem Cell and Developmental Biology, Genome Institute of Singapore, Biopolis, Singapore, Singapore; 3 BasAM, Norwegian School of Veterinary Science, Oslo, Norway; Radboud University Nijmegen, The Netherlands

## Abstract

**Background:**

Uncovering epigenetic states by chromatin immunoprecipitation and microarray hybridization (ChIP-chip) has significantly contributed to the understanding of gene regulation at the genome-scale level. Many studies have been carried out in mice and humans; however limited high-resolution information exists to date for non-mammalian vertebrate species.

**Principal Findings:**

We report a 2.1-million feature high-resolution Nimblegen tiling microarray for ChIP-chip interrogations of epigenetic states in zebrafish (*Danio rerio*). The array covers 251 megabases of the genome at 92 base-pair resolution. It includes ∼15 kb of upstream regulatory sequences encompassing all RefSeq promoters, and over 5 kb in the 5′ end of coding regions. We identify with high reproducibility, in a fibroblast cell line, promoters enriched in H3K4me3, H3K27me3 or co-enriched in both modifications. ChIP-qPCR and sequential ChIP experiments validate the ChIP-chip data and support the co-enrichment of trimethylated H3K4 and H3K27 on a subset of genes. H3K4me3- and/or H3K27me3-enriched genes are associated with distinct transcriptional status and are linked to distinct functional categories.

**Conclusions:**

We have designed and validated for the scientific community a comprehensive high-resolution tiling microarray for investigations of epigenetic states in zebrafish, a widely used developmental and disease model organism.

## Introduction

The advent of chromatin immunoprecipitation (ChIP) combined with ChIP DNA hybridization to microarrays (ChIP-chip) or high-throughput sequencing (ChIP-seq) has enabled genome-wide mapping of post-translational histone modifications and binding sites for transcription factors and chromatin regulators in a variety of mammalian cell types [1]–[7]. Among histone post-translational modifications (PTMs) examined, trimethylation of H3 lysine 4 (H3K4me3) marks the promoter of most genes; in contrast, H3K27me3 is associated with a fraction of promoters of inactive or weakly expressed genes within a facultative heterochromatin environment [1], [3]. Interestingly, embryonic stem cells harbor chromatin domains co-enriched in H3K4me3 and H3K27me3, which contain transcriptionally ‘poised’ developmentally-regulated genes [1]. Upon differentiation, activated genes undergo H3K27 demethylation while retaining H3K4me3, whereas silenced genes retain H3K27me3 and may (or may not) be demethylated on H3K4 [1], [4], [8]. Co-enrichment in H3K4me3 and H3K27me3 has been proposed to constitute a mark of priming for transcriptional activation (or repression) in embryonic stem cells, a concept since extended to various somatic progenitor cell types [4], [6], [9], [10].

Genome-wide epigenetic state maps are also being built in non-mammalian species such as *Drosophila melanogaster* and *Xenopus laevis* primarily because of their attractiveness in developmental studies. H3K4 methylation has been detected by ChIP-chip on promoter regions of *Drosophila* embryos [11] along with, in cultured cells, regions marked by methylated H3K27 [12], [13] (see also [14]). In addition, maps of H3K4me3- and H3K27me3-enriched regions have been established by ChIP-chip and ChIP-seq in *Xenopus* with the aim of improving 5′ end gene annotation and establishing the spatial environment of methylated H3K4 and H3K27 domains in developing embryos [15], [16].

Epigenomic information has also started to emerge in zebrafish (*Danio rerio*). A custom-made promoter array covering −1.5 kb to +0.5 kb relative to the transcription start site (TSS) at ∼250 base-pair (bp) intervals has initially been used to map H3K4me3 binding sites in embryos [17]. Representation by at least two probes was required for a region to be included on the array. The design included 11,117 promoter regions which owing to redundancy in the genome assembly mapped to 12,545 locations in the genome [17]. More recently, a NimbleGen 385,000-probe array covering ∼31 megabase (mb) of the zebrafish genome with a median ∼80 bp resolution was used for the determination of H3K4me3, H3K27me3, H3K36me3 and RNA polymerase II enrichment sites before and after zygotic gene activation [18]. The tiled regions included selected developmentally-regulated genes and two contiguous regions on chromosomes 3 and 11, collectively including 685 RefSeq genes.

We report here a high-density 2.1-million probe array design covering ∼20 kb of upstream regulatory regions and exons of all 12,697 RefSeq zebrafish genes, at a median 92-bp resolution. We used these arrays to map regions of trimethylated H3K4 and H3K27 over >251 mb of the genome, or 17.5% of the Zv8 genome assembly, in the embryo-derived fibroblast ZF4 cell line [19]. The data notably uncover developmentally regulated genes marked by H3K4me3 and H3K27me3 despite the differentiated state of ZF4 cells. The array constitutes a robust platform for epigenetic investigations in zebrafish, a widely used developmental and disease model organism.

## Results

### Zebrafish High-density Promoter Array Design

**Figure 1:**
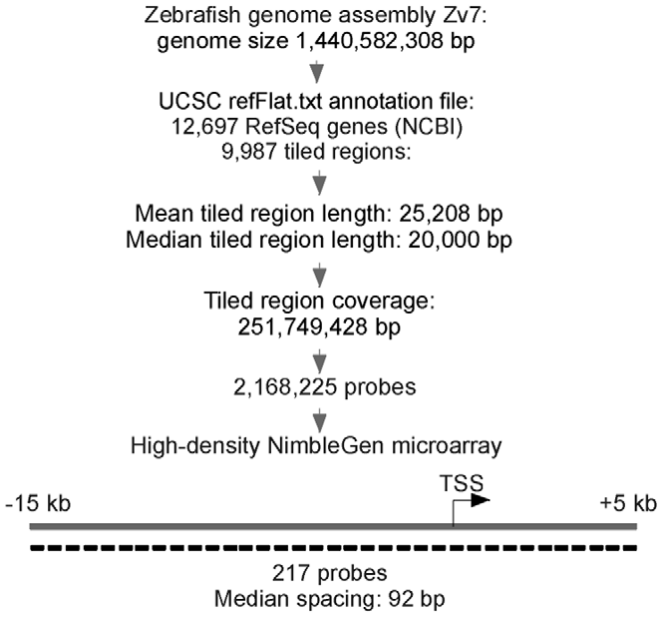
Design of the zebrafish ChIP-chip tiling array. Array probe design (upper chart) and median promoter coverage within the tiled regions, shown for the positive strand. Probes are represented by the dashed lines. Spacing between dashes symbolizes probe spacing. Note: the array was designed from the Zv7 assembly as the Zv8 assembly was unpublished at the time. Zv7 reported a genome size of 1,440,582,308 bp; Zv8 currently reports a genome size of 1,441,241,298 bp.

The zebrafish Zv8 assembly (www.sanger.ac.uk/Projects/D_rerio) reports 1,441,241,298 bp with 24,147 protein-coding genes, of which 12,697 are RefSeq genes. To tile all zebrafish promoters at high resolution, we designed a high-density microarray (Figure 1). Tiled regions on the array were defined based on the UCSC refFlat.txt annotation file from the Zv7 assembly, Zv8 being unpublished at the time. This file only covers NCBI genes; thus the array contains 12,697 RefSeq genes, corresponding to 14,836 RefSeq transcripts, and covers the upstream regulatory regions (including promoters) of all 14,836 transcripts. The array contains 2,168,225 isothermal probes with an average probe length of 55 bp, encompassing 9,987 tiled regions with a mean tiled region length of 25,208 bp (median length 20,000 bp). Median probe spacing is 92 bp and total tiled region coverage is 251,749,428 bp, representing 17.5% of the Zv8 zebrafish genome size. Genes are evenly divided between the positive and negative DNA strands, with approximately 2% mapping to both strands, mostly due to transcripts mapped to two or more places in the genome (data not shown). The array design is available under GEO accession number GSE23872 and the array is available from the supplier.

### Profiling of H3K4me3 and H3K27me3 on Promoters in Zebrafish Fibroblasts

**Figure 2:**
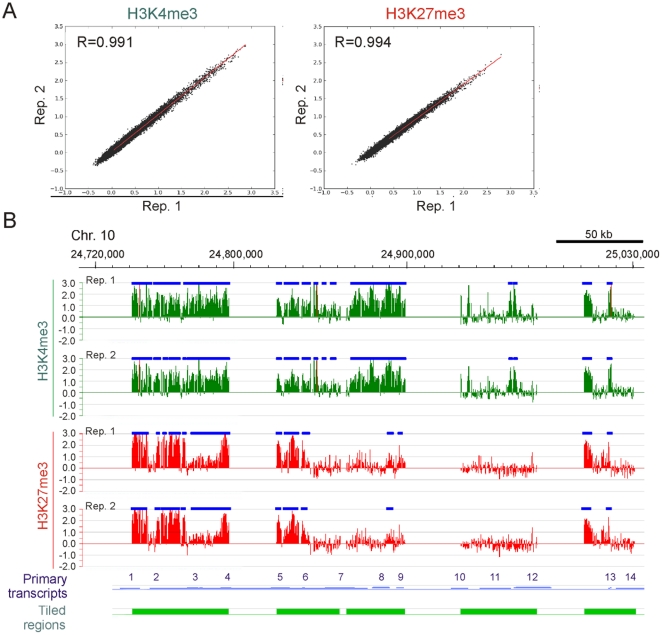
Reproducibility of zebrafish ChIP-chip experiments. (A) Two-dimensional scatter plots of MaxSixty values for H3K4me3 and H3K27me3 log_2_ signal intensities detected in each of two ChIP-chip replicates from ZF4 cells. Correlation coefficient (R) and regression line are shown. (B) H3K4me3 and H3K27me3 profiles detected by ChIP-chip in two independent replicates through 310 kb of zebrafish chromosome 10. Data are expressed as log_2_ ChIP/input ratios. Position of methylation peaks are shown as blue horizontal bars. Tracks representing primary transcripts and tiled regions are also shown. Primary transcripts included in the region are as follows: 1) *sin2*; 2) NM_001003421; 3) NM_200663; 4) NM_001008616; 5) ENSDART00000081978; 6) *ripply3*, 7) ENSDART00000081992; 8) *dyrk1aa*; 9) ENSDART00000058411; 10) ENSDART00000088605; 11) NM_001037708; 12) *hyou1*; 13) *hist2h2l*; 14) *znf259*. Red bars in the H3K4me3 tracks indicate probes with out-of-scale signal intensity.

**Figure 3:**
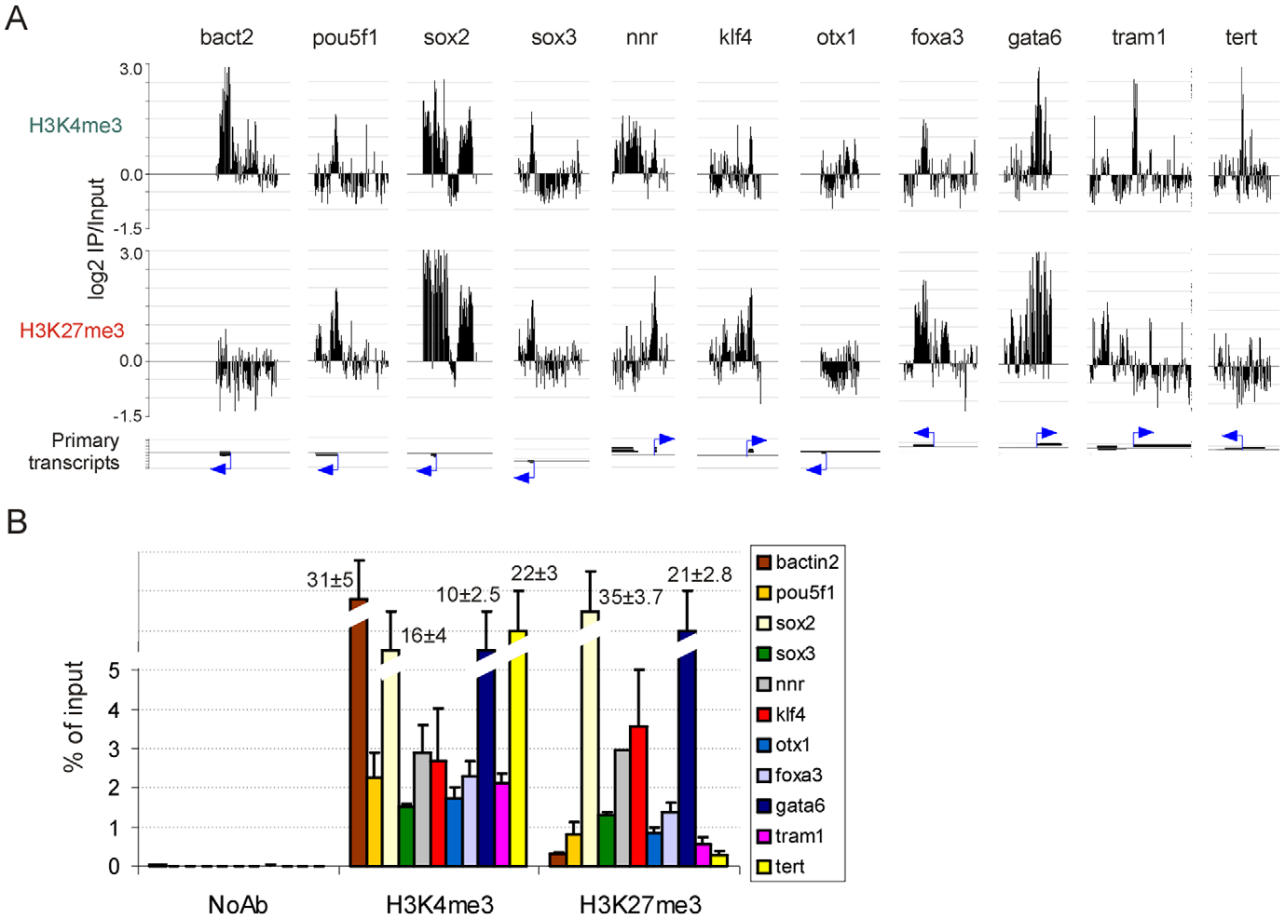
Quantitative PCR validation of ChIP-chip data. (A) ChIP-on-chip profiles of H3K4me3 and H3K27me3 enrichment on indicated genes. Position of primary transcripts and TSS (arrow) are shown. (B) ChIP-qPCR analysis of H3K4me3 and H3K27me3 enrichment on the same genes as in (A) from separate duplicate ChIPs. ChIP DNA was not WGA amplified prior to PCR. Position of amplicons and primer sequences for each gene are shown in Table S3. Note the correlation between ChIP- chip and ChIP-qPCR data.

Chromatin from ZF4 cell cultures was subjected to 3-4 independent H3K4me3 and H3K27me3 ChIPs. Consistency in the amount of DNA precipitated by each antibody was determined by spectrophotometry (data not shown). ChIP DNA from two replicates was amplified, labeled and hybridized to the tiling arrays. To establish the reproducibility of the ChIP-chip replicates, correlation analysis of log_2_ ChIP/Input ratios between replicates was done with values resulting from MaxSixty calculations. This algorithm was derived from the MaxFour algorithm [20] which we and others have previously extended to MaxTen calculations [21], [22]. MaxSixty scores each gene by finding the highest average log_2_ ratio among 60 consecutive probes per tiled region. Robust reproducibility between replicates was demonstrated by two-dimensional scatter plots for H3K4me3 and H3K27me3 (correlation coefficient R>0.99; Figure 2A). MaxTen applied to these arrays also showed strong correlations between replicates (R = 0.988 for H3K4me3 and R = 0.987 for H3K27me3; data not shown). Reproducibility was also shown by the similarity of enrichment profiles within tiled regions and by the similarity of the mapping of detected peaks (see below), as exemplified in Figure 2B. Additional validation of the ChIP-chip data was provided by the similarity of H3K4me3 and H3K27me3 enrichment profiles detected on the arrays (Figure 3A) and by qPCR from non-amplified ChIP DNA (Figure 3B) (see also [23]).

**Figure 4:**
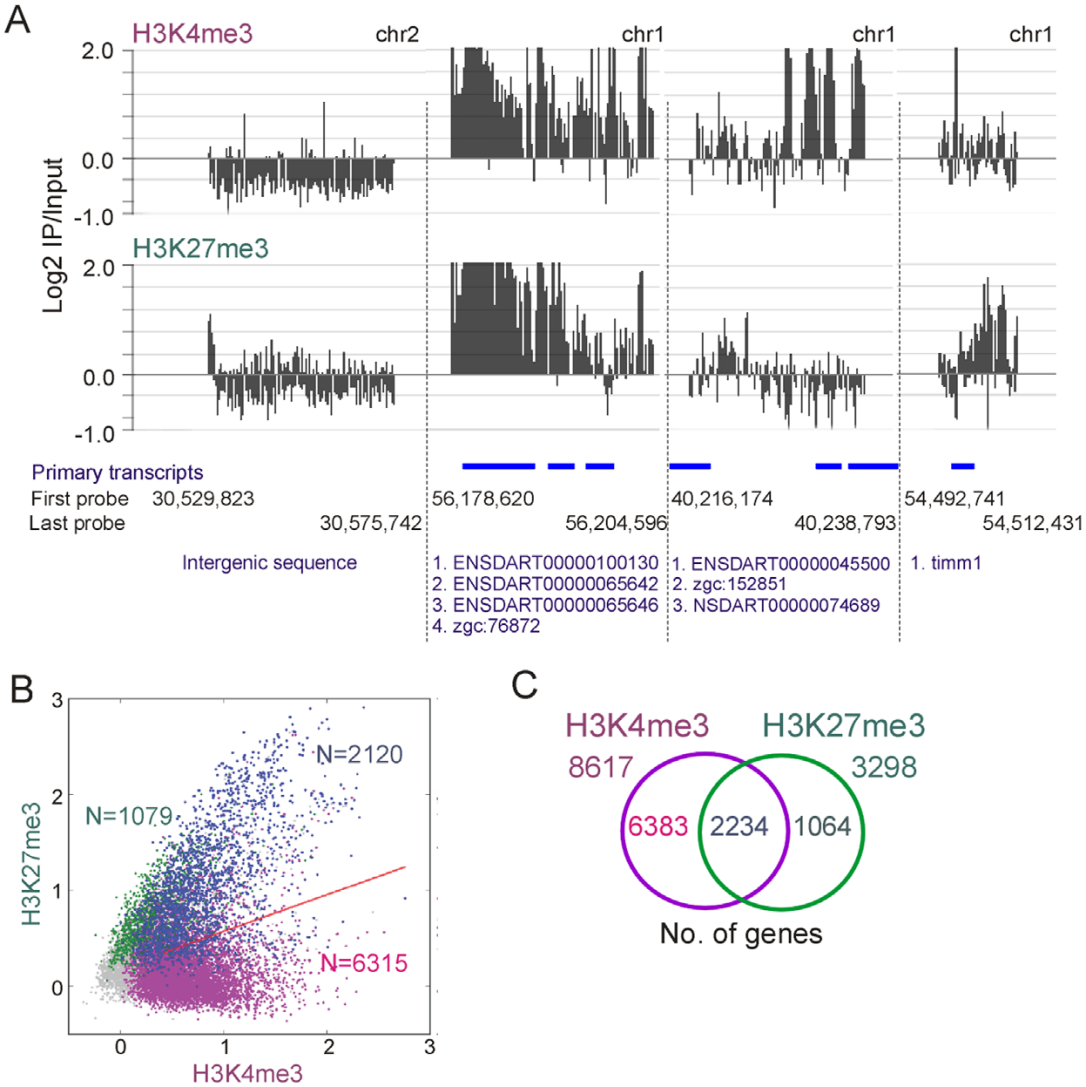
H3K4me3 and H3K27me3 enrichment profiles in ZF4 cells. (A) Distinct H3K4me3 and H3K27me3 enrichment profiles on indicated genomic regions. Genomic positioning is indicated by nucleotide number of the first (5′) and last (3′) probe in the tiled region. Gene names or accession numbers as well as their genomic position are shown in blue. (B) 2-D scatter plot of averaged MaxSixty values for H3K4me3 vs. H3K27me3 log_2_ signal intensities. Data points (all points being shown in gray) were colored to visualize classification according to peak calling highlighting H3K4me3-enriched promoters (purple; N = 6315), H3K27me3-enriched promoters (green; N = 1079) and H3K4me3/K27me3-co-enriched promoters (blue; N = 2120). Red line is the regression line through all data points. (C) Venn diagram analysis of H3K4me3 and H3K27me3 genes.

Two-dimensional scatter plots of MaxSixty values for H3K4me3 vs. H3K27me3 log_2_ signal intensities for all promoters showed distinct enrichment patterns for each of these modifications, supporting profiles evidenced by SignalMap scanning (Figures 4A and 4B). Using a peak detection algorithm with a false discovery rate (FDR) of ≤0.1 for each modification, we identified a total of 8435 H3K4me3-enriched promoters and 3199 H3K27me3-enriched promoters (Figure 4B). Among those, we also identified 2120 promoters co-enriched in H3K4me3 and H3K27me3 (Figure 4B; blue data points), suggesting that regions co-enriched in these modifications exist in zebrafish, or non-exclusively, that distinct cell populations in the ZF4 culture harbor either modification on these regions.

Because in our analysis genes with TSSs located close to each other were mapped to the same promoter region, the actual number of genes with a promoter enriched in either modification was higher than the number of promoters identified. Applying the peak detection algorithm with FDR≤0.1 to genes, we found 8617 genes with an H3K4me3-enriched promoter and 3298 genes with an H3K27me3-enriched promoter; these are subsequently referred to as ‘H3K4me3 genes’ and ‘H3K27me3 genes’, respectively (Figure 4C). We also identified 2234 ‘H3K4me3/K27me3 genes’ which made up 26% of all H3K4me3 genes and 68% of all H3K27me3 genes. These numbers were in the range of those shown in earlier studies of mouse and human cells despite differences in cell types, detection methods and peak calling algorithms [1], [3]–[6], [22], [24].

### H3K4 and H3K27 Trimethylation Occupancy Profiles on Promoters and on 5′ End of Coding Regions

**Figure 5:**
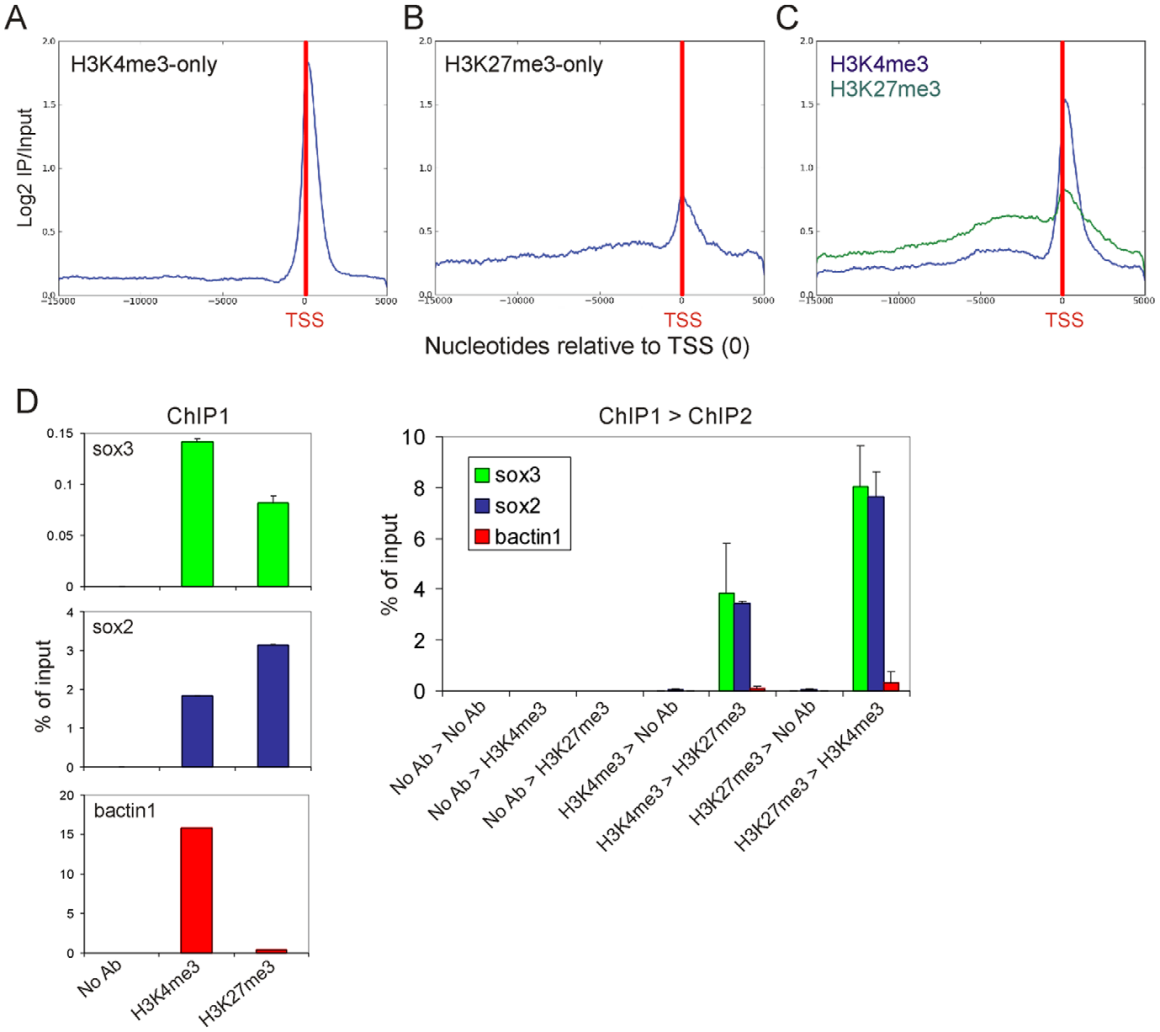
Distribution of H3K4me3 and H3K27me3 on promoters. Metagene analysis of the distribution of H3K4me3 and H3K27me3 occupancy on (A) H3K4me3-only, (B) H3K27me3-only and (C) H3K4me3/K27me3 tiled regions, relative to the TSS (red vertical bar). (D) Sequential ChIP analysis of H3K4me3 and H3K27me3 co-enrichment on the *sox3*, *sox2* promoters and on *bactin1*, downstream of the coding region. Panels on the left show results from the first ChIP using antibodies indicated on the x-axis. The graph on the right shows results of the re-ChIP experiment as indicated on the x-axis.

To assess the average enrichment profiles of H3K4 and K27 trimethylation and the overlap of these modifications, we computed a composite metagene for the collection of genes enriched in one or both modifications over the tiled regions. On H3K4me3-only genes, H3K4me3 displayed no enrichment over the first ∼14 kb but showed marked enrichment starting at ∼1 kb upstream of the TSS and extending, yet decreasing, over ∼2 kb into the coding region (Figure 5A). Maximal enrichment was detected at nucleosome +1 after the TSS. On H3K27me3-only genes, H3K27me3 displayed wider enrichment on either side of the TSS than H3K4me3 on H3K4me3-only genes, but lower maximal enrichment (Figure 5B). Moreover, in contrast to H3K4me3, maximal H3K27me3 level occurred exactly over the TSS, indicating a modified chromatin arrangement at the TSS in genes harboring trimethylated H3K4 or H3K27 only. Over regions detected with both modifications, H3K4me3 and H3K27me3 showed a slightly higher level particularly over the 5 kb upstream of the TSS, relative to on “-only” regions, suggesting that H3K4me3 and H3K27me3 co-enriched promoters harbor a distinct chromatin configuration.

Recent sequential ChIP data from blastula-stage zebrafish embryos have provided evidence for H3K4me3- and H3K27me3 co-enrichment on a proportion of genes in this species [18], raising the possibility that at least some of these genes may be H3K4me3/H3K27me3 ‘bivalent’. To assess this possibility in our study, we carried out sequential H3K4me3→H3K27me3 ChIP, and *vice versa*, using a recently improved protocol [18]. Figure 5D indicates that *sox3* and *sox2* were co-enriched in both H3K4me3 and H3K27me3 regardless of which modification was immunoprecipitated first (right panel), supporting the single ChIP data (Figure 5D, left panels; see also Figure 3). This was in contrast to *bactin1* which is only enriched in H3K4me3 (Figure 5D). The sequential ChIP assay, therefore, suggests H3K4me3/K27me3 bivalency on the *sox2* and *sox3* promoters.

### Trimethylation of H3K4 or H3K27 Delineates Genes Associated with Distinct Functional Categories

**Figure 6:**
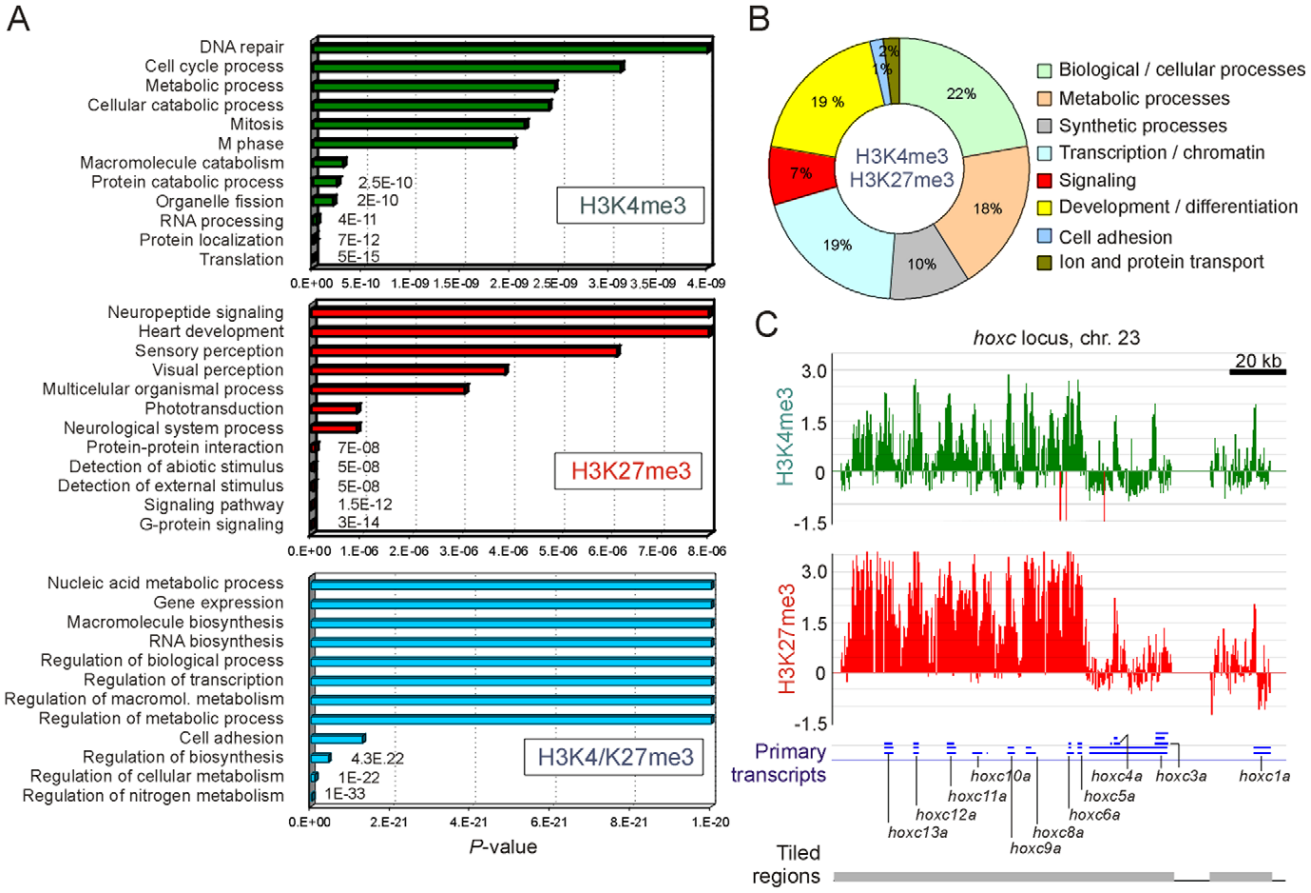
Genes marked by H3K4me3 and/or H3K27me3 are linked to distinct functional GO terms. (A) GO term enrichment of genes containing H3K4me3, H3K27me3 or H3K4/K27me3 promoters in ZF4 cells. The twelve most significant GO terms are shown as a function of significance from bottom (highest significance) to top. (B) Representation of all enriched GO terms among H3K4/K27me3 genes. All enriched GO terms are listed in Table S1. (C) H3K4me3 and H3K27me3 enrichment profiles on the developmentally regulated *hoxc* locus, expressed as log_2_ ChIP/Input (y axis).

To address the biological significance of enrichment in trimethylated H3K4 or H3K27 revealed by ChIP-chip, we identified Gene Ontology (GO) terms enriched among these genes. We found enrichment in distinct terms for genes harboring either or both marks (Figure 6A; Table S1). Enriched GO terms of H3K4me3 genes were related to translation and protein processing, catabolic and metabolic processes and cell cycle control. GO terms for H3K27me3 genes included signal transduction functions and included transcription factors implicated in developmental and organismal processes. H3K4me3/K27me3 genes were enriched in metabolic and synthetic processes, and in developmentally-regulated transcription, chromatin regulation, signaling and developmental functions (Figure 6A,B; Table S1). The latter pertained primarily to early embryo and fetal development (including genes of the four *hox* loci; Figure 6C and data not shown) and to ectodermal differentiation with emphasis on neuronal differentiation (Table S1). A large number of genes involved in signaling, chromatin and transcription regulation were also developmentally regulated. The functional groups identified here in the ZF4 zebrafish cell line are thus overall similar to those reported earlier in other cell types such as mouse or human embryonic stem cells [1], [3], [5], somatic progenitors [6], [9] and differentiated cells [2], [25], albeit on different sets of genes. Association with developmentally-regulated genes seems therefore to be a feature of H3K4me3/K27me3 co-enrichment, regardless of species or cell type; however, we also found association of trimethylated H3K4 and H3K27 with metabolic processes, which to our knowledge has not been reported earlier.

### H3K4 and H3K27 Trimethylation in Relation to Gene Expression

**Figure 7:**
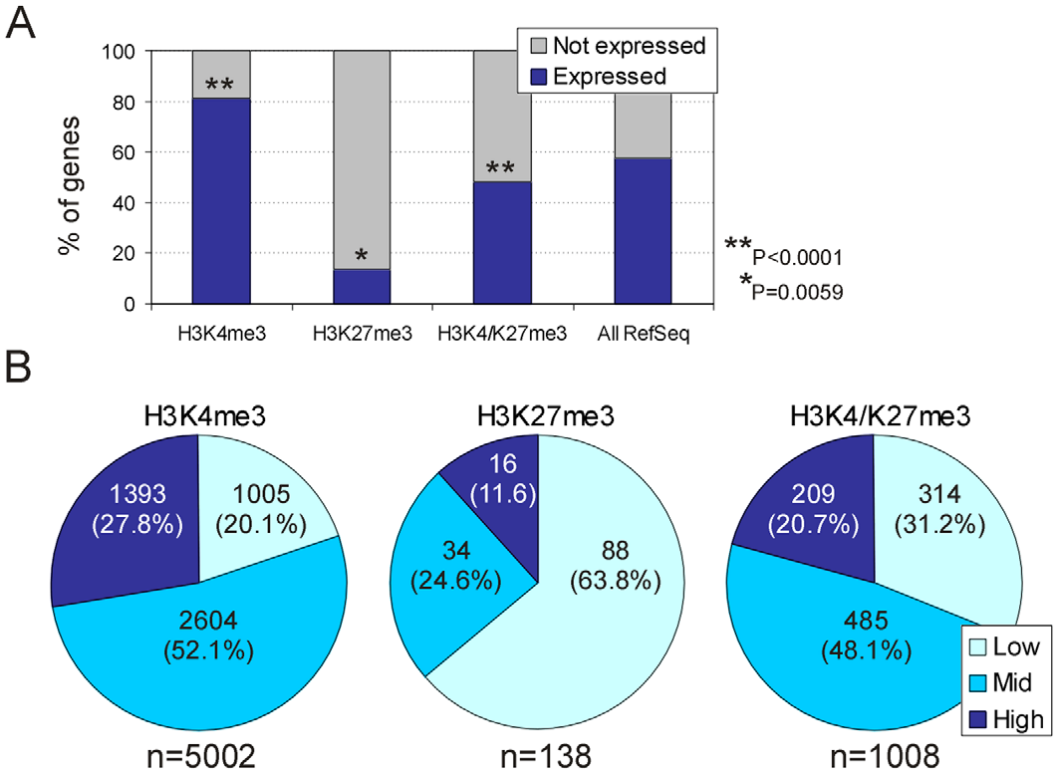
Gene expression status in relation to histone modification enrichment. (A) Percentage of expressed and non-expressed genes marked by indicated histone modifications, and among all RefSeq genes co-represented on both the Agilent and Nimblegen arrays (n = 11,971). (B) Percentile analysis of expression levels of genes marked by indicated modifications. Low, 25^th^ percentile; Mid, 25^th^<x<75^th^ percentile; High, >75^th^ percentile. Only expressed genes (blue bars in A) are taken into the analysis. Numbers within the pie charts indicate the number of genes in each category; numbers are the bottom indicate the number of expressed genes (Present call) marked by either or both histone modifications.

We next assessed the proportion of expressed genes carrying trimethylated H3K4 and/or H3K27 using an Agilent expression microarray platform by, first, defining present (expressed) and absent (not expressed) calls. We found that 81% of H3K4me3 genes were expressed, representing an enrichment over the proportion of expressed Refseq genes identified in ZF4 cells regardless of the associated histone modification (57%; *P*<10^−4^ relative to the proportion of expressed RefSeq genes; Chi-square with Yates' correction; Figure 7A). In contrast, the vast majority of H3K27me3 genes was not expressed (84%; *P* = 0.005), while 48% of H3K4me3/K27me3 genes (*P*<10^−4^) were expressed (Figure 7A). Percentile analysis of expression levels indicated that 80% of expressed H3K4me3 genes showed high (>75^th^ percentile; 28% of genes) or moderate (25^th^–75^th^ percentile; 52% of genes) expression while 20% were only weakly expressed (25^th^ percentile; Figure 7B). In contrast, expression of the majority (64%) of H3K27me3 genes was at low level. Moreover, a significant proportion (48%) of H3K4me3/K27me3 genes was moderately expressed, however H3K4me3/K27me3 genes also harbored a greater proportion of weakly expressed genes than H3K4me3-only genes, at the expense of strongly expressed genes (*P*<10^−4^, relative to H3K4me3; Figure 7B). Therefore, as in other species or cell types examined [1], [3], [5], [18], there is in cultured zebrafish cells a relationship between expression status, assessed by mRNA detection or H3K36me3 occupancy on exons [18], and histone modifications associated with the corresponding genes. While the vast majority of expressed genes are marked by H3K4me3, trimethylation of H3K27 is mainly associated with the promoter of repressed genes, but can also mark a proportion of weakly expressed genes when they are co-enriched with H3K4me3.

Enriched GO terms and corresponding gene list among expressed and non-expressed genes marked by H3K4me3, H3K27me3 or by both modifications are provided in Table S2. In particular, repressed H3K4me3/K27me3 genes included genes with transcriptional regulatory and signaling functions involved in development (Table S2). For instance, all developmentally-regulated H3K4me3/K27me3-coenriched *hox* genes, except three (*hoxb5b*, *hoxc5a* and *hoxc9a*) were not expressed (Table S2). H3K4me3/K27me3 genes that are expressed, though at low level, were enriched in cell adhesion, macromolecule metabolism functions and included transcriptional regulators and cofactors (Table S2). Interestingly, expressed genes with chromatin remodeling functions (although marked by H3K4/K27me3) included the HP1 homologs *cbx1a* and *cbx1b*, zinc-finger protein *znf703*, ring-finger protein *rnf168*, nucleosome assembly protein 1-like 1 *nap1l1*, chromobox homolog 4 *cbx4* and the Polycomb repressor complex components *bmi1* and *eed* (Table S2). Of note, two additional Polycomb genes expressed in ZF4 cells, *ezh2* and *suz12*, were enriched in H3K4me3 only.

## Discussion

The advent of comprehensive genome-wide tools for analyzing chromatin states and binding of transcription regulators has provided significant advances in our understanding of gene regulatory networks in many systems. Although ChIP-seq can provide full genome coverage, ChIP-chip approaches remain valuable for analysis of focused regions. Epigenetic states have been mapped by ChIP-chip in primarily mouse, man, *Drosophila* and *Arabidopsis* (e.g., [1], [5], [7], [12], [21], [26], [27]). In zebrafish, only recently have promoter arrays been reported for a few hundreds of RefSeq genes [18], or for most RefSeq genes but at low resolution [17]. These have proven useful to identify novel H3K4me3-enriched regions, highlighting putative novel promoters [17], and for mapping epigenetic transitions during development [18]. We now extend these studies and report a high-density array covering 251.7 mb of the zebrafish genome at 92-bp resolution.

We identify over 8600 genes enriched in H3K4me3 and 3300 genes enriched in H3K27me3. These numbers are in the range of those reported earlier in mice and humans, arguing for robust genome coverage of the modified histones immunoprecipitated. Moreover, we find that in addition to the 52% of H3K4me3/K27me3 genes, 19% of genes marked by H3K4me3 only are not expressed. This figure is reminiscent of the 28% of inactive genes recently found to be marked by H3K4me3 only in blastula-stage zebrafish embryos [18]. GO term analysis reveals that these genes encode ribosomal proteins, molecules involved in protein metabolism, as well as histone H2A and H3 isoforms (Table S2). How genes enriched in H3K4me3-only are repressed remains to be determined. In light of the complexity of combinations of modified histones that decorate the genome, it is unlikely that these genes are “monovalent” as referred to previously [18] because monovalency would imply that they are not enriched in any other histone mark. Repression of these genes may involve low levels of H3K27me3 below the peak detection limit, co-enrichment in di- or trimethylated H3K9, co-enrichment in other repressive histone marks, or non-exclusively, DNA methylation. Similar arguments may account for the transcriptionally active state of H3K27me3-only genes. These are also unlikely to exist in a monovalent state and may be co-occupied by a level of H3K4me3 below peak detection, together with other non-assessed transcriptionally permissive modifications.

Whether genomic regions are effectively co-enriched in a combination of trimethylated H3K4 and H3K27, as suggested by sequential ChIP experiments in mouse embryonic stem cells [1], in zebrafish embryos [18] and in this study, remains debated [15], [18]. The proportion of H3K4me3/K27me3 genes identified in ZF4 cells, the proportion of transcriptionally inactive H3K4me3/K27me3 genes, and the functional categories of H3K4me3/K27me3 genes determined by GO annotation, are consistent with those reported in mouse and human embryonic stem cells [1], [3]–[5], [8], mouse blastocysts [7] and zebrafish embryos [18]. It seems therefore that H3K4me3/K27me3 co-enrichment represents a signature of developmentally- or differentiation-regulated genes. Nonetheless, the geographic distribution of these marks relative to one another needs to be considered when interpreting occupancy profiles on linearized genomes averaged from cell populations. Spatial differences and fluctuations in the deposition of these marks are indeed likely to occur, based on mosaic gene expression patterns observed in cell cultures [28] and during embryonic development [15].

Average enrichment profiles of H3K4me3 and H3K27me3 over promoter regions evidenced in our metagene analysis are in agreement, in terms of enrichment level and width of occupancy, with recent ChIP-chip data from zebrafish embryos [18]. This observation provides additional validation of our arrays. A peak of occupancy over the 5′ end of gene bodies (TSS) is therefore a consistent feature of these histone marks regardless of species, cell type or mode of detection (array, sequencing or qPCR). Nevertheless, a noticeable difference in the distribution of H3K4me3 among species or cell types examined to date lies in the detection of a “dip” immediately upstream of the TSS in mouse and human cells [7], [10], [22], [29]. This dip is not apparent in the *Xenopus* ChIP-chip or ChIP-seq data [15], [16], nor in the zebrafish ChIP-chip data [17], [18] (this study). This difference is unlikely to be due to array design, in particular probe spacing, because that is very similar (80–92 bp) in the mouse [7], human [10] or zebrafish [18] (this study) arrays used. It is also probably not due to fundamental differences in chromatin preparation because mouse, human and zebrafish chromatin was cross-linked with formaldehyde, extracted under similar salt concentrations, and fragmented by sonication. Similar dips in acetylated H3K9 and H4K16 occupancy have also notably been detected in the mouse [30]. Such dips have initially been interpreted as nucleosome depletion at the TSS of transcribing genes [29], and have subsequently been interpreted to result from differential biochemical extraction of histones during chromatin preparation for ChIP in these regions of nucleosome instability [14], [31]. Detectable H3K4me3 enrichment at the TSS in *Xenopus* and zebrafish [15]–[18] (this study) irrespective of chromatin preparation and buffers used in these studies, suggests differences in nucleosome composition in this region between fish and mammals. It may also lie in differences in representation of the profiles. Another possibility may involve the bioinformatic collapse of TSSs close to each other which we have performed in our metagene computations, in particular when single transcription units contain multiple TSSs under one gene. Alternatively, the difference in H3K4me3 enrichment at the TSS could be due to potential variability introduced by less robust prediction of TSSs in RefSeq genes in zebrafish, for which genes are still being annotated. All these possibilities remain to be explored in future studies.

The array reported here currently represents, to our knowledge, the highest genomic coverage and probe density available for studies in zebrafish. In addition to covering all RefSeq promoters, the array also notably spans the entire coding region of 42% of the genes represented on the array. This feature will most likely prove useful for uncovering at high resolution fluctuations in epigenetic states on gene regulatory and coding regions during development, in disease models and developmental mutants, or in the context of exon splicing [32].

## Materials and Methods

### Cells and Antibodies

The ZF4 zebrafish embryo-derived fibroblast cell line (American Type Culture Collection; www.atcc.org) [19] was cultured in DMEM/F2 Glutamax buffered with bicarbonate, 15% fetal bovine serum, 50 µg/ml Gentamicin and 40 µg/ml bovine pancreas insulin, in a humidified atmosphere of 5% CO_2_ at 28°C. Antibodies to H3K4me3 were from Diagenode (cat# pAb-003-050; www.diagenode.com) and antibodies to H3K27me3 were from Millipore (cat# 07-449; www.millipore.com).

### Expression Microarray Analysis

RNA was isolated from batches of ∼10^6^ cells using the RNeasy Mini Kit (Qiagen; www.qiagen.com). RNA samples were stored at −80°C until processed for microarray. Microarray techniques were according to Agilent's One-Color Microarray-Based Gene Expression Analysis (Quick Amp Labeling) manual Version 5.7 (Agilent; www.agilent.com). Briefly, ∼400 ng total RNA and spike-in RNA were used to prepare cDNA and Cy3-labeled cRNA. Labeled cRNA was fragmented prior to hybridization. Arrays were hybridized and washed as per Agilent's protocol. Arrays used contained 44K probes representing all known genes and one probe for each non-redundant UniGene clusters. Arrays also contained 153 different negative control probes. Array format was 4x 44K and was custom-designed by Agilent. For analysis of gene expression in the context of histone modifications, only genes represented on the Agilent and Nimblegen arrays were considered.

Arrays were quantitated using GenePix (http://www.moleculardevices.com). Data were background-corrected and normalized using quantile normalization with the Limma package from Bioconductor (http://www.bioconductor.org). Probes were then scored for detection of expression in each experiment. A probe was assigned a ‘Present’ score if (Mean(FPI) - Mean(BPI)) / StDev(BPI) was equal to or greater than two, where FPI is the set of foreground pixel intensities, and BPI is the set of background pixel intensities. Probes received an ‘Absent’ score otherwise. Genes were assigned Present/Absent calls as follows: gene G received a ‘Present’ score if a majority of probes from the replicate experiments corresponding to G scored Present; G was scored Absent otherwise. Average gene expressions were then calculated for each gene by taking the median probe intensity for all probes intensities in the replicate experiments that corresponded to each gene. Gene expression quantiles were calculated by determining the 0th, 25th, 75th and 100th percentile for the set of genes which scored as ‘Present’ in the detection test above. Microarray expression data are MIAME compliant and have been deposited in a MIAME compliant database (GEO accession number GSE23872).

### Chromatin Immunoprecipitation

To prepare chromatin, ZF4 cells (15×10^6^) were harvested, washed in PBS and crosslinked in suspension for 8 min in 1% formaldehyde in PBS containing 20 mM Na-butyrate. Cross-linking was stopped by adding glycine to 125 mM. Cells were washed twice by gentle vortexing in 0.5 ml PBS/20 mM Na-butyrate and centrifugation at 400 g in a swing-out rotor for 10 min at 4°C. The supernatant was removed and cells were lysed by thorough vortexing in 300 µl lysis buffer (50 mM Tris-HCl, pH 8.0, 10 mM EDTA, 1% SDS, protease inhibitors and 20 mM Na-butyrate) and sonication on ice for 8×30 sec, with 30-sec pauses on ice in-between, using a Sartorius Labsonic M sonicator with a 3-mm diameter probe at setting 0.5 cycle and 30% power (Sartorius; www.sartorius.com) to an average fragment length of 400 bp, assessed by agarose gel electrophoresis. Samples were centrifuged at 12,000 g for 10 min at 4°C and the supernatant (chromatin) was diluted in RIPA ChIP buffer (10 mM Tris-HCl, pH 7.5, 140 mM NaCl, 1 mM EDTA, 0.5 mM EGTA, 1% Triton X-100, 0.1% SDS, 0.1% Na-deoxycholate, protease inhibitors, 20 mM Na-butyrate) to 2 U A_260_ before immunoprecipitation.

Chromatin in 100 µl RIPA ChIP buffer was mixed with 10 µl antibody-Dynabeads Protein A (Invitrogen) complexes overnight at 4°C. To generate these complexes, 2.4 µg primary antibody was pre-mixed with Dynabeads Protein A for 2 h at 4°C in RIPA buffer. The immunoprecipitated ChIP material was washed three times in RIPA buffer and once in 10 mM Tris-HCl, pH 8.0, 10 mM EDTA buffer. Cross-link was reversed, proteins digested and DNA eluted in a single step for 2 h at 68°C in 150 µl ChIP elution buffer (20 mM Tris-HCl, pH 7.5, 5 mM EDTA, 20 mM Na-butyrate, 50 mM NaCl, 1% SDS and 50 µg/ml proteinase K) containing 5 µg RNase (Roche; www.roche.com). After magnetic separation, beads were re-eluted with another 150 µl ChIP elution buffer for 15 min and both eluates were pooled. Eluted DNA was purified by phenol-chloroform isoamylalcohol extraction and ethanol precipitation using 10 µl acrylamide as carrier and dissolved in MilliQ H_2_O. ChIP and input DNA was amplified using the Whole Genome Amplification WGA4 kit (Sigma-Aldrich; www.sigmaaldrich.com), omitting cell lysis and DNA fragmentation steps. Amplification products were cleaned up using the QiaQuick PCR Purification Kit (Qiagen) and eluted in 30 µl elution buffer diluted ten times (Qiagen). After DNA purification, samples were quantified by NanoDrop (NanoDrop Technologies; www.nanodrop.com). WGA4-amplified DNA (5 µl) were used for quantitative (q)PCR validation. ChIPs were done from at least four independent chromatin preparations, from which two were sent to NimbleGen.

### Sequential ChIP

Sequential H3K4me3→H3K27me3 and H3K27me3→H3K4me3 ChIPs were performed essentially as described [18]. The first immunoprecipitation was performed with 150 µl of 2 A_260_ units chromatin using 5 µl H3K4me3 or H3K27me3 antibody (H3K4me3 Diagenode pAB-003-050 and H3K27me3, Millipore 07-449) covalently coupled to 8 µl tosyl-activated beads (Invitrogen). Immunoprecipitated chromatin was eluted in 130 µl sequential ChIP elution buffer (0.1% SDS, 50 mM NaHCO_3_) for 20 min at 37°C. Twenty microliters of the eluate were used for analysis of the first ChIP by qPCR. For the second round of ChIPs, the remaining 100 µl were divided in two, diluted 10 times in RIPA buffer and ChIPs carried out with relevant bead-only negative controls. The re-ChIP DNA was eluted with ChIP elution buffer as described in the single ChIP protocol above, and ChIP samples were analyzed by qPCR.

### Quantitative PCR

ChIP DNA was analyzed before whole genome amplification by duplicate qPCR on a MyiQ Real-time PCR Detection System using IQ SYBR® Green (BioRad; www.biorad.com) [23]. Sequences of ChIP primers used are shown in Table S3. PCR conditions were 95°C for 3 min and 40 cycles of 95°C for 30 sec, 60°C for 30 sec and 72°C for 30 sec.

### DNA Labeling and Promoter Array Hybridization

ChIP and input DNA fragments were labelled with Cy5 and Cy3, respectively, and hybridized onto Nimblegen custom-designed zebrafish promoter arrays (www.nimblegen.com) (see Figure 1). Arrays covered the upstream regulatory sequences, including promoters, of all 12,697 zebrafish RefSeq genes, ranging from −15,000 to +5,000 bp relative to the TSS. Probes consisted of 2.17 million 55-mers (range 55-70-mers) tiled throughout non-repetitive sequences at a median spacing of 92 bp. Sequence source for probes was the zebrafish Zv7 assembly (www.sanger.ac.uk/Projects/D_rerio) as the Zv8 assembly was not published at the time. However, all references to numbers of genes or nucleotides covered are to the Zv8 assembly. ChIP and input DNA labeling, hybridization and detection were performed at NimbleGen. ChIP-chip data are MIAME compliant and have been deposited in a MIAME compliant database (GEO accession number GSE23872).

### ChIP-chip Data Analysis

Signal intensity data were extracted from scanned images using the NimbleScan software. Log_2_ ChIP/Input ratios were scaled and centred around zero by subtracting the bi-weight mean for the log_2_ ratio values for all features on the array from each log_2_ ratio value. Peaks were detected by searching for four or more probes with a signal above a cut-off value using a 500-bp sliding window. Cut-off values were a percentage of a hypothetical maximum defined as (mean +6[standard deviation]). Ratio data were randomized 20 times to evaluate the probability of false positives, and each peak was assigned a false discovery rate (FDR) score. Normalization and peak detection were performed by Nimblegen as per their protocols (http://www.nimblegen.com/products/chip/data_guide.html). This process uses a cut-off range of 90% to 15%, with higher cut-offs corresponding to more stringent peak detection, as reflected in the FDR. The Nimblegen protocol was evaluated as part of a study that objectively analyzed the performance of a number of commercially available ChIP-chip array platforms and detection algorithms [33], and found to deliver reliable results.

### Metagene Computation

Metagene analysis was performed essentially as described [17]. Genes with a high probability of enrichment (FDR ≤0.10) in H3K4me3 or H3K27me3 within the tiled region were used to assemble a metagene of average composite binding. Each region was interrogated for probes and these were mapped into a 20-kb wide window at the appropriate offsets based on strand orientation. Linear interpolation was used to estimate the fold enrichment at each base position within the window. This interpolation left the 5′ and 3′ ends of the window under-represented. The metagene was created by calculating the mean of the values mapped to each position by all the regions found enriched in either or both modifications. If the offset corresponded to the exact location of a probe within a specific tiled region, values were directly measured; if not, values were linearly interpolated from the values of the two flanking probes [17]. Genes enriched by only one mark were selected from the entire set of genes harboring the mark and then removing from that set all genes also possessing a peak for the other mark. Genes enriched in both modifications were selected from the entire set of genes harboring either mark.

### Gene Ontology Analysis

Gene ontology (GO) term enrichments within a target gene set were calculated using Bioconductor GOstats [34]. GOstats identifies functional terms for selected genes and provides a significance of enrichment for a term by giving a *P*-value indicating the probability that the identified term is enriched among the target genes relative to what would be expected by chance based on the number of genes in the genome that belong to this term.

## Supporting Information

Table S1 **GO terms enriched among genes marked by H3K4me3, H3K27me3 or both marks.**
(XLS)

Table S2 **Enriched GO terms with gene list among expressed and non-expressed genes marked by H3K4me3, H3K27me3 or both marks.**
(XLS)

Table S3 **ChIP-qPCR primers used in this study.**
(DOC)
