# Supplementary material for: Tiling Histone H3 Lysine 4 and 27 Methylation in Zebrafish Using High-Density Microarrays
Source: PLoS One. 2010 Dec 20;5(12):e15651. doi: 10.1371/journal.pone.0015651 (PMC3004928; doi:10.1371/journal.pone.0015651)
Supplement: Table S3 — ChIP-qPCR primers used in this study. (DOC) [file pone.0015651.s003.doc]

**Tiling Histone H3 Lysine 4 and 27 Methylation in Zebrafish Using High-density Microarrays**

Leif C. Lindeman^1*^, Andrew H. Reiner^1*^, Sinnakaruppan Mathavan^2^, Peter Aleström^3^ and Philippe Collas^1§^

^1^Institute of Basic Medical Sciences, Faculty of Medicine, University of Oslo, and Norwegian Center for Stem Cell Research, 0317 Oslo, Norway, ^2^Stem Cell and Developmental Biology, Genome Institute of Singapore, Biopolis, Singapore, ^3^BasAM, Norwegian School of Veterinary Science, Oslo, Norway.

*These authors contributed equally.

^§^Email: philippe.collas@medisin.uio.no

**Supporting Information**

**Table S3.** ChIP-qPCR primers used in this study

| Gene | Forward primer (F) 5’🡪3’  Reverse primer (R) 5’🡪3’ | Position rel. to TSS (nt) | Annealing temp. (^o^C) |
| --- | --- | --- | --- |
| *bactin1* | F: gcattggtaactgtaataatct  R: gctgtgtctgtaactgta | +3750/+3593 | 60 |
| *bactin2* | F: actatgaactgaaccgactg  R: ctgcgatcaattacacaacc | +393/+1072 | 60 |
| *foxa3* | F: ggacttctcaggattatacttg  R: aggctcttctgttatcttct | -324/-208 | 60 |
| *gata6* | F: taggacattacaagacgacacaa  R: atgaaggcattagtttctcacaag | -734/-643 | 60 |
| *klf4* | F: atctgataggctacaactac  R: tggctggatgtctacc | -113/-17 | 60 |
| *nnr* | F: actgtaaagggcctcgaaca  R: cctccctcatttgctgttgt | -189/-63 | 60 |
| *otx1* | F: gatgttctacctgtggattg  R: ctaagagcgtaggacacc | -127/-26 | 60 |
| *pou5f1* | F: gatacacctcgcgttcccaaacatgtc  R: ttgctaatcaatcggagttggaggcag | -188/-38 | 60 |
| *sox2* | F: tgctgaccgtccgtaacc  R: acaaccattcatagagcgactg | -141/-28 | 60 |
| *sox3* | F: tgttatgctgtcaatccaaatcac  R: gctcaaactctggtcaagtaaag | -148/+3 | 60 |
| *tram1* | F: actgaccagcgacctttttg  R: gtcgtgcccaaaaattcatc | -511/-384 | 60 |
| *tert* | F: gcgtgtcgttatctgggagt  R: cgtgcttggggagttctatt | -69/-10 | 60 |
